# Syndecan-1 and FGF-2, but Not FGF Receptor-1, Share a Common Transport Route and Co-Localize with Heparanase in the Nuclei of Mesenchymal Tumor Cells

**DOI:** 10.1371/journal.pone.0007346

**Published:** 2009-10-05

**Authors:** Fang Zong, Eleni Fthenou, Nina Wolmer, Péter Hollósi, Ilona Kovalszky, László Szilák, Carolin Mogler, Gustav Nilsonne, Georgios Tzanakakis, Katalin Dobra

**Affiliations:** 1 Department of Laboratory Medicine, Division of Pathology, Huddinge University Hospital, Karolinska Institutet, Stockholm, Sweden; 2 Department of Histology, Division of Morphology, School of Medicine, University of Crete, Heraklion, Greece; 3 1st Institute of Pathology and Experimental Cancer Research, Semmelweis University Budapest, Hungary; 4 Department of Pathology, University of Heidelberg, Heidelberg, Germany; Universität Heidelberg, Germany

## Abstract

Syndecan-1 forms complexes with growth factors and their cognate receptors in the cell membrane. We have previously reported a tubulin-mediated translocation of syndecan-1 to the nucleus. The transport route and functional significance of nuclear syndecan-1 is still incompletely understood. Here we investigate the sub-cellular distribution of syndecan-1, FGF-2, FGFR-1 and heparanase in malignant mesenchymal tumor cells, and explore the possibility of their coordinated translocation to the nucleus. To elucidate a structural requirement for this nuclear transport, we have transfected cells with a syndecan-1/EGFP construct or with a short truncated version containing only the tubulin binding RMKKK sequence. The sub-cellular distribution of the EGFP fusion proteins was monitored by fluorescence microscopy. Our data indicate that syndecan-1, FGF-2 and heparanase co-localize in the nucleus, whereas FGFR-1 is enriched mainly in the perinuclear area. Overexpression of syndecan-1 results in increased nuclear accumulation of FGF-2, demonstrating the functional importance of syndecan-1 for this nuclear transport. Interestingly, exogenously added FGF-2 does not follow the route taken by endogenous FGF-2. Furthermore, we prove that the RMKKK sequence of syndecan-1 is necessary and sufficient for nuclear translocation, acting as a nuclear localization signal, and the Arginine residue is vital for this localization. We conclude that syndecan-1 and FGF-2, but not FGFR-1 share a common transport route and co-localize with heparanase in the nucleus, and this transport is mediated by the RMKKK motif in syndecan-1. Our study opens a new perspective in the proteoglycan field and provides more evidence of nuclear interactions of syndecan-1.

## Introduction

Proteoglycans (PGs) are highly sulfated macromolecules, whose protein cores bear covalently attached glycosaminoglycan (GAG) chains. Cell surface heparan sulfate proteoglycans (HSPGs) are present in most cells of both vertebrates and invertebrates. At the cell surface the GAG chains interact with many ligands such as growth factors (GFs), cytokines, adhesion molecules etc. [1], [2], and they are essential modulators of cellular signaling in embryonic development and tumorigenesis [3], [4]. The transmembrane HSPG syndecan-1 is the prototype member of the syndecan family, and it participates in assembling signaling complexes by presenting GFs to growth factor receptors (GFRs) [5]. The ability of basic fibroblast growth factor (FGF-2) to bind to fibroblast growth factor receptor-1 (FGFR-1) has been proven to depend largely on the presence of heparan sulfate (HS), which interacts with both FGF-2 and FGFR-1, stabilizing the ligand/receptor complex [6], [7], [8], [9], [10].

The HS chains can be degraded by heparanase through enzymatic cleavage [11] and in this way the HSPG-bound GFs can be liberated. Experimental studies show that cleavage of the HS chain may generate oligosaccharide sequences, which can either inhibit or potentiate the effect of the GFs [12]. Notably, HS is not only a substrate for, but also a regulator of heparanase uptake [13], and syndecan-1 in turn is able to regulate the biological activity of heparanase [14].

Traditionally, syndecan-1 has been thought to exert its effect in signaling at the level of the cell membrane. However, we have previously shown a regulated nuclear translocation and co-localization of syndecan-1 with tubulin in the mitotic spindle [15]. We detected prominent nuclear syndecan-1 not only in malignant mesothelioma but also in various adenocarcinomas and in neuroblastoma cells. Similar but weaker nuclear staining was seen in different benign cells of mesenchymal origin [15]. This was the first evidence for the nuclear translocation of the whole syndecan-1 molecule. The HS chains of PGs have long been known to be present in the nuclear compartment of various normal and transformed cells concurrently with inhibition of cell growth [16], [17], [18], [19]. Apart from syndecan-1, other HSPGs can also be present in the nucleus [20], e.g. syndecan-2 [21], [22] and glypican-1 [23].

The route and functional significance of this nuclear transport of syndecan-1 is still incompletely understood. Mounting evidence suggests a similar nuclear accumulation of GFs [24], [25], and their receptors [26], [27], [28]. Exogenously added FGF-2 has been shown to internalize and translocate to the nucleus in proliferating cells, whereas in quiescent cells it remains mainly cytoplasmic [29]. The nuclear and nucleolar translocation of FGF-2 and FGFR-1 occurs around the restriction point of the cell cycle in mid-late G1 phase, suggesting a controlled nuclear entry [30], [31]. Moreover, the efficiency of the nuclear FGF-2 translocation is increased in the presence of heparin [32].

In our previous work, double staining experiments clearly demonstrated that syndecan-1 is structurally linked to the intracellular microtubule system in all phases of cell division, and that inhibition of microtubule polymerization by vinblastine treatment hampers the nuclear translocation of syndecan-1 [15]. As syndecan-1 can bind both GFs and their GFRs, we aimed in this study to investigate the possibility of a regulated co-translocation of syndecan-1, FGF-2 and FGFR-1. Furthermore, we hypothesize that the syndecan-1/tubulin complex may not only act as a vehicle for the transport of GFs to the cell nucleus, but it may also constitute a functional entity in an intracrine route, which operates independently from the cell surface receptor function. Our experiments are designed to clarify the translocation of the syndecan-1/FGF-2/FGFR-1 complex, and the structural requirement for the nuclear transport of syndecan-1.

## Materials and Methods

### Cell lines and cell culture conditions

Three cell lines of mesenchymal origin were used in this study. The STAV malignant mesothelioma cells were generally grown in RPMI 1640 medium containing 25 mM HEPES (GIBCO, Grand Island, NY, USA) and 2 mM L-Glutamine. The STAV-AB cell sub-line was supplemented with 10% human AB serum and displayed epithelial differentiation, while the STAV-FCS cell sub-line was supplemented with 5% fetal bovine serum (FBS) and 5% calf serum (CS) to achieve a fibroblast-like morphology [33]. The B6FS human fibrosarcoma cells [34] were grown in RPMI 1640 + GlutaMAX™-1 (GIBCO) supplemented with 10% FBS and 20 µg/ml Gentamicin (GIBCO). All cells were cultured in 75 cm^2^ Tissue Culture Flasks (Sarstedt, Newton, NC, USA) in humidified 5% (v/v) CO_2_ at 37°C and culture medium was changed twice a week. The STAV-AB cells show a low endogenous syndecan-1 expression level on the cell surface [35], while the B6FS cells do not express syndecan-1 [36].

### Sub-cellular localization of syndecan-1, endogenous FGF-2, heparanase and FGFR-1

The sub-cellular distributions of syndecan-1, heparanase, FGF-2 and FGFR-1 were examined by immunocytochemical analysis and subsequent confocal laser microscopy. Cells were seeded onto Superfrost Plus microscope slides and allowed to adhere for 6–48 hours before they were fixed with 3% paraformaldehyde at 37°C for 10 minutes, and then permeabilized with 0.1–0.5% Triton X-100 (Sigma, Steinheim, Germany) at 37°C for 15 minutes. For the tubulin depolymerization experiments, cells were fixed in ice-cold methanol for 10 minutes, rehydrated in PBS and taken directly for immunocytochemistry.

For visualization of cell membrane syndecan-1 and HS reactivity, the cells were not permeabilized in order to keep the integrity of the cell membrane. Non-specific binding was blocked with 3% goat serum (DAKO A/S, Giostrup, Denmark) for 30 minutes; thereafter the primary antibody was added (Table 1). Slides were incubated overnight in a humidified chamber at 4°C, followed by 30 minutes of incubation with fluorescent secondary antibodies (Table 2) in darkness at room temperature. The slides were counterstained with 1 mg/L Bisbenzimide H33342 (FLUKA, Steinheim, Germany), and mounted in DAKO Fluorescent Mounting Medium (DAKO, Via Real Carpiteria, CA, USA).

**Table 1:** Primary antibodies used.

No.	Antigen	Clone	Dilution	Company/catalogue no.
1	Syndecan-1(CD138), mouse monoclonal IgG1	B-B4	1∶4	Serotec MCA681 H
2	Syndecan-1, goat polyclonal	-	1∶20	Santa Cruz sc-7099
3	FGF-2, mouse monoclonal IgG2a	MC-GF1	1∶4	Serotec MCA1400G
4	FGF-2, goat polyclonal	N-19	1∶20	Santa Cruz sc-1390
5	FGFR-1, mouse monoclonal IgM	VBS1	1∶20	Biogenesis
6	HS, mouse monoclonal IgM	10E4	1∶200	Seikagaku 370255
7	Heparanase, mouse monoclonal	mAB 130	1∶400	InSight Ltd.
8	Heparanase, rabbit polyclonal	pAB 733	1∶10	Vlodavsky *et. al.*
9	α-tubulin, mouse monoclonal IgG1	B-5-1-2	1∶2000	Sigma T5168

**Table 2:** Secondary antibodies used.

No.	Preparation	Dilution	Company/catalogue #
I	Goat α-mouse IgG (H+L), F(ab')_2_ Alexa 568	1∶800	Molecular Probes A11019
II	Donkey α-goat IgG (H+L) Alexa 568	1∶800	Molecular Probes A11057
III	Goat α-mouse IgG (H+L), F(ab')_2_ Alexa 488	1∶800	Molecular Probes A11017
IV	Goat α-mouse IgM Alexa 488	1∶800	Molecular Probes A21042
V	Goat α-mouse IgM Alexa 568	1∶800	Molecular Probes A21043
VI	Goat α-mouse IgG_1_ Alexa 568	1∶800	Molecular Probes A21124
VII	Goat α-rabbit IgG (H+L) highly cross-adsorbed, Alexa 488	1∶800	Molecular Probes A11034
VIII	Goat α-mouse IgG (H+L) highly cross-adsorbed, Alexa 568	1∶1600	Molecular Probes A11031

Blocking solutions consisted of 3% goat or donkey serum, or 1% BSA in PBS.

Double labeling was performed by simultaneous incubation with the respective primary antibodies. Negative controls were used, either with affinity purified mouse or goat IgG (Table 1) or, in the case of CD138, by preincubating the antibody with the syndecan-1 epitope.

A large number of primary and secondary antibody combinations were tested and the corresponding isotype controls were always included to allow background subtraction. We performed single label experiments to exclude over-bleeding between the channels.

### Nuclear complex co-immunoprecipitation and immunoblotting

Nuclear complex co-immunoprecipitation (Co-IP) was carried out using the Nuclear Complex Co-IP Kit (Active Motif Europe, Rixensart, Belgium) according to the manufacturer's instructions. Briefly, nuclear extracts of sub-confluent cells were prepared 24–36 hours after seeding of cells stably transfected with syndecan-1/EGFP constructs or those transfected with just EGFP vector. Protein concentrations were determined by the Bradford method using bovine serum albumin (BSA) as a standard. For Co-IP, 100 µg of each nuclear extract was reacted with 3 µg of goat polyclonal antibody against the C-terminus of syndecan-1 (C-20, sc-7099, Santa Cruz Biotechnology, Inc., Santa Cruz, CA, USA). The coupled protein/antibody complexes were adsorbed onto protein G Sepharose™ Fast Flow beads (GE Healthcare Life Sciences, Uppsala, Sweden). The protein/antibody complex beads were re-suspended in TBS buffer. The immunoprecipitated proteins were released from the beads by boiling at 95–100°C for 3–5 minutes and spinning briefly to collect supernatants. The nuclear precipitates from equal amounts of control and sample proteins were blotted directly to a nitrocellulose membrane, using the Minifold II Slot Blot System (Schleicher & Schuell, Inc., Keene, NH, USA.), and followed by immunoblotting with a mouse anti-human FGF-2 monoclonal antibody (MCA1400G, AbD Serotec Ltd., Oxford, UK). The secondary antibody was an ECL™ Peroxidase-labeled anti-mouse antibody (NA931VS, GE Healthcare Life Sciences, Uppsala, Sweden). Chemiluminescence detection was performed using Western Lightening ™ Chemiluminescence Reagent Plus (NEL 104, Perkin Elmer LAS, Inc., Waltham,,MA, USA). Chemiluminescence signals were recorded with a charge-coupled device camera (FluorChem®SP, AlphaInnotech, San Leandro, CA, USA).

### Confocal laser microscopy

For detailed visualization of the distribution of antibody reactivity, confocal laser microscopy was used. This was performed with a Leica TCS NT confocal laser scanning microscope equipped with an ArKr laser, permitting the detection of signals from the fluorochromes with emission wavelengths at 488 nm and 568 nm. Scanning was performed using a 63×1.2 NA objective lens and higher magnifications with zoom function. Images were obtained by scanning in the XY direction with a focal depth of 0.3 µm and were then processed with Adobe Photoshop software. For each experiment the excitation and sensitivity of the detector were adjusted so that the signals were normalized to the corresponding negative controls. The instrument settings were adjusted to omit non-specific reactivity and over-bleeding.

### Effect of drugs interfering with microtubule assembly and cell cycle progression on the sub-cellular distribution of syndecan-1, FGF-2 and FGFR-1

Vinblastine promotes depolymerization of tubulin and its redistribution into paracrystalline inclusion bodies. To depolymerize tubulin structures, cells were exposed to 10 µM of vinblastine (Velbe® - Eli Lilly Sweden AB, Stockholm, Sweden) for 2 hours, as an established way to demonstrate tubulin-dependent transport [37]. This was followed by immunocytochemical staining for FGF-2, syndecan-1 (CD 138) and FGFR-1. All experiments were performed in at least triplicate. Double staining was performed for tubulin (mouse monoclonal IgG1, detected by goat anti-mouse IgG (H+L), F(ab')_2_ Alexa 488) and FGF-2 (goat polyclonal, detected by donkey anti-goat IgG (H+L) Alexa 568), or syndecan-1 (goat polyclonal, detected by donkey anti-goat IgG (H+L) Alexa 568). Double staining was also performed with FGFR-1 (mouse monoclonal IgM, detected by goat anti-mouse IgM Alexa 488) and tubulin (mouse monoclonal IgG1, detected by goat anti-mouse IgG_1_ Alexa 568).

Doxorubicin is a chemotherapeutic agent known to cause cellular damage via a number of mechanisms including inhibition of topoisomerase II, nucleotide intercalation, free radical formation and inhibition of DNA replication. Doxorubicin treatment results in G2 arrest and interferes with cell cycle progression by sustaining the G2 arrest after DNA damage [38]. Mesothelioma cells were exposed to 1.2 µg/ml Doxorubicin (Adriamycin®, Pharmacia & Upjohn, Stockholm, Sweden) for 48 hours, followed by immunocytochemical staining with mouse monoclonal IgG1 against syndecan-1, detected by goat anti-mouse IgG (H+L), F(ab')_2_ Alexa 488.

### Sub-cellular localization of exogenously added FGF-2 in the mesothelioma cells

The sub-cellular fate of exogenously added, fluorescence-tagged FGF-2 was monitored *in vitro*. For this purpose recombinant human FGF-2 (R&D Systems, Minneapolis, MN, USA) was labeled with Alexa Fluor® 488 (A10235, Molecular Probes, Inc., Leiden, The Netherlands), according to the instructions of the manufacturers. Based on our previous data on the mitogenic effect of FGF-2 on mesothelioma cells, the concentration of FGF-2 used ranged from 5–50 ng/mL. The observation times were 16 h, 24 h and 42 h after seeding. In parallel, double staining was performed for syndecan-1 (goat polyclonal, detected by donkey anti-goat IgG (H+L) Alexa 568) and the endogenous FGF-2 (mouse monoclonal IgG2a, detected by goat anti-mouse IgG (H+L), F(ab')_2_ Alexa 488).

### Transfection of cells with full-length and truncated syndecan-1 constructs and detection of nuclear accumulation of syndecan-1

To further clarify the possible role of syndecan-1, we have transfected the cells with a syndecan-1/EGFP construct, or a truncated variant, coding only for the RMKKK sequence, which corresponds to a hypothesized nuclear localization signal of syndecan-1. The syndecan-1/EGFP constructs were prepared by Szilák Labor Ltd, (Szeged, Hungary) and the pEGFP-N1 vector, used as a negative control, was purchased from BD Biosciences, (Clontech, Palo Alto, CA, USA). The construct of human syndecan-1 was cloned on a HindIII – BamHI fragment into pEGFP-N1 plasmid in-frame with the N-terminal end of EGFP. The correct DNA sequence of this construct was verified by DNA sequencing. The plasmids were amplified in *E. coli* and purified with EndoFree Plasmid Maxi Kit (QIAGEN GmbH, Hilden, Germany). Their purity was determined by spectrophotometry and agarose gel electrophoresis.

Cells were transfected with the constructs above, using Effectene Transfection Reagent (QIAGEN GmbH, Hilden, Germany). Optimization of the transfection was carried out according to the manufacturer's guidelines. Briefly, about 2×10^5^ cells were seeded into 6-well plates and incubated for 24 hours to reach 40–80% of confluence at the time of transfection. Transient transfections were performed using 0.4 µg DNA and a DNA/Effectene ratio of 1∶25. The presence of the functional syndecan-1/EGFP fusion protein was verified by immunocytochemical analysis with antibodies against syndecan-1 (CD-138) and HS (mouse monoclonal IgM, clone 10E4, Seikagaku Corporation, Tokyo, Japan) using confocal microscopy, as described above. The appearance and sub-cellular distribution of the newly synthesized syndecan-1 were evaluated at various time points (6, 12, 24, 48, and 72 hours) after transfection using a fluorescence microscope (Leica DM IRBE, Openlab 3.0.4 software) to follow the EGFP-positive cells.

### Site directed mutational analysis of the RMKKK sequence

The RMKKK sequence of syndecan-1 corresponding to Arg - Met –Lys – Lys - Lys was subjected to site directed mutagenesis by GenScript Corporation (NJ, USA) to generate three mutants. These were mutant 1, Ala - Met - Leu - Lys – Lys (AMLKK); mutant 2, Ala - Met - Lys - Leu – Lys (AMKLK); and mutant 3, Arg – Met -Leu - Leu – Lys (RMLLK).

Cells were transfected with the RMKKK construct, or the mutants 1–3, according to the transfection protocol described above, and their fate was subsequently followed by confocal laser microscopy.

## Results

### Sub-cellular localization of syndecan-1, heparanase, FGF-2 and FGFR-1

**Figure 1:**
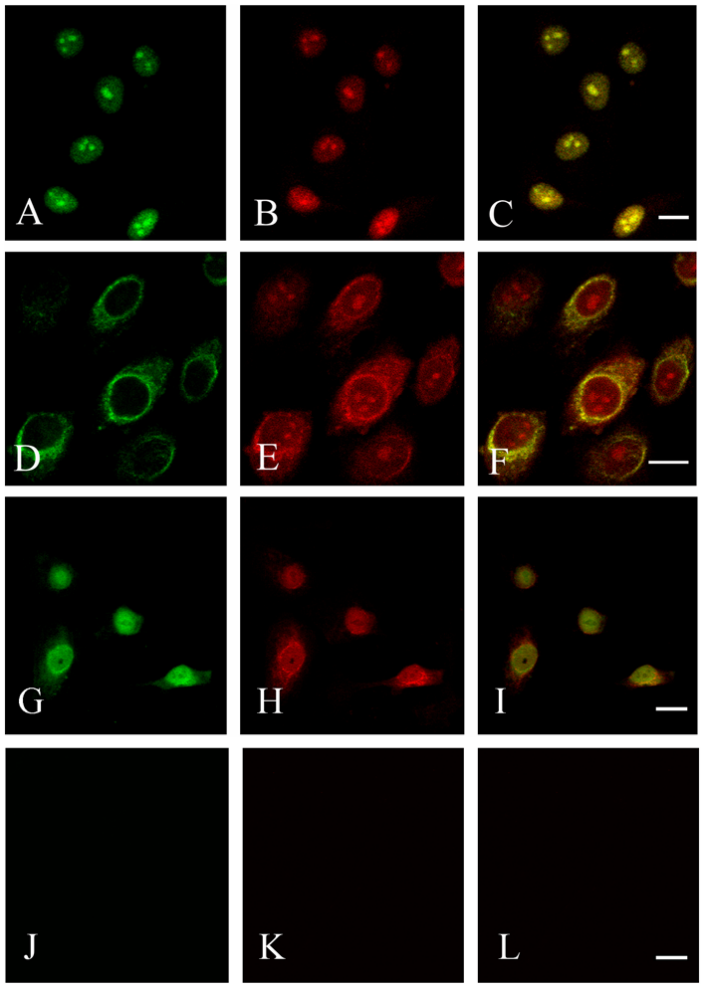
FGF-2 and heparanase, but not FGFR-1 co-localize with syndecan-1 in the nuclear compartment of mesothelioma cells. Immunocytochemical staining of the STAV-AB cells for FGF-2 (A) or syndecan-1 (B) showed distinct nuclear and nucleolar localization. Merged images revealed a complete co-localization of FGF-2 and syndecan-1 (C) inside the nuclear envelope. FGFR-1 showed prominent perinuclear staining (D), and syndecan-1 (E) showed only a partial co-localization with FGFR-1 in the perinuclear area (F). Strong nuclear reactivity was also detected for heparanase (G), and syndecan-1 (H), with a clear nuclear co-localization (I). The corresponding isotype controls are shown as (J), (K) and (L). *Bar*  = 10* µ*m. Antibodies used: (A) 3+ III. (B) and (E) 2+ II. (D) 5+ IV. (G) 8+ VII. (H) 1+I (Tables 1 and 2).

The sub-cellular distributions of syndecan-1, heparanase, FGF-2 and FGFR-1 were examined by immunocytochemistry. Syndecan-1 showed maximal intensity in the cell nuclei, nucleoli and/or in the perinuclear area (**Figure 1B, E and H**, respectively). The relative distribution of syndecan-1 between these locations, however, varied somewhat. Apart from the nuclear reactivity, cytoplasmic and cell membrane staining at the cell-cell contact sites were also clearly detected in malignant cells (**Figure 1** **, Figure S1**). Double staining with antibody specific for FGF-2 revealed similar distinct nuclear and nucleolar reactivities (**Figure 1A**). The nuclear reactivity of FGF-2 and syndecan-1 strictly co-localized (**Figure 1C**). Also heparanase (**Figure 1G**) showed co-localization with syndecan-1 in the nucleus (**Figure 1H, I**), suggesting the presence of a functional entity. In contrast, FGFR-1 (**Figure 1D**), known to form signaling complexes with FGF-2, was found only in the cytoplasm with maximal intensity in the perinuclear area, where it co-localized with syndecan-1 (**Figure 1E, F**). Notably, there was no FGFR-1 reactivity present in the nucleus of mesothelioma cells. The staining intensity at these locations varied, but the staining pattern was reproducible. The cells only displayed background levels of fluorescence when stained with corresponding isotype controls (**Figure 1J-L**).

**Figure 2:**
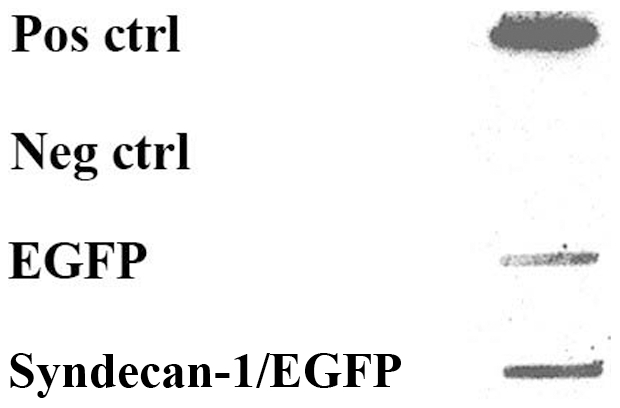
Syndecan-1 and FGF-2 co-immunoprecipitate in the nucleus of the STAV-AB cells. Co-IP and immunoblotting were performed on nuclear extracts of sub-confluent cultures of the STAV-AB cells, stably transfected with syndecan-1/EGFP construct or EGFP vector, as described in “Materials and Methods”. Nuclear extract (100 µg) was incubated with 3 µg of syndecan-1 antibody, or with no antibody (negative control). The nuclear precipitates from equal amounts of control and sample proteins were slot-blotted onto a nitrocellulose membrane and then probed with antibody to FGF-2. A crude nuclear extract from syndecan-1/EGFP transfected cells was used as positive control. Co-IP using a specific antibody to syndecan-1 also pulled down FGF-2, as compared to the negative control. The amount of FGF-2 was higher in nuclear extracts from syndecan-1 overexpressing cells, compared to the EGFP control.

Crude nuclear extracts from these cells also showed immunoreactivity to both syndecan-1 and FGF-2. The latter was increased considerably, following upregulation of syndecan-1 by transfection (data not shown). When syndecan-1 was specifically immunoprecipitated, FGF-2 was also found in the precipitate. The amount of co-precipitated nuclear FGF-2 increased when syndecan-1 was overexpressed (**Figure 2**), demonstrating that syndecan-1 is needed for this nuclear translocation.

### Effect of drugs interfering with microtubule assembly and cell cycle progression on the nuclear translocation of syndecan-1

**Figure 3:**
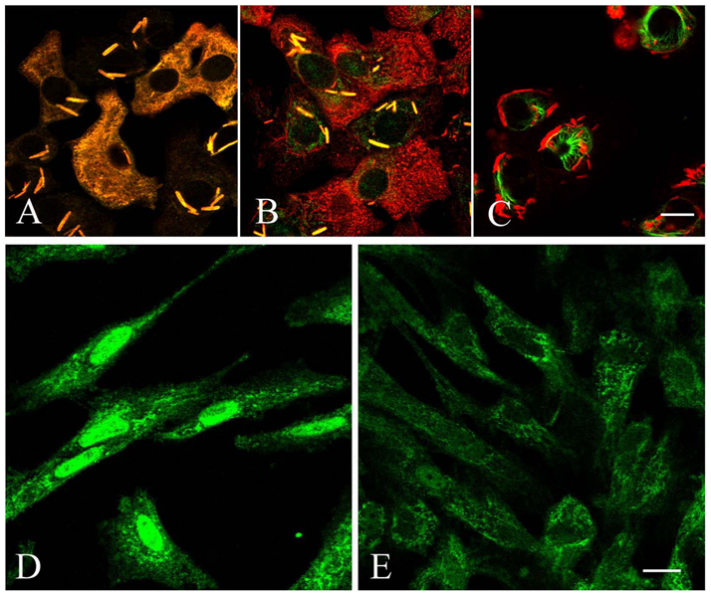
Effects of drugs interfering with microtubule assembly and cell cycle progression. The sub-cellular localizations of FGF-2 (A), syndecan-1 (B) and FGFR-1 (C) in the STAV-AB cells were detected after depolymerization of tubulin by vinblastine shown as co-localized paracrystalline structures (*yellow crystals*). Double staining was performed with tubulin (*green*) and either FGF-2 (*red*) (A) or syndecan-1 (*red*) (B), and both revealed strict co-localization of these components (*yellow crystals*, A, B). No co-localization of FGFR-1 (*green*) and tubulin (*red*) is detected (C), indicating a different transport route. Untreated STAV-FCS mesothelioma cells showed strong nuclear reactivity for syndecan-1 (D). Doxorubicin almost completely inhibited the nuclear transport of syndecan-1 in these cells (E).

Treatment with vinblastine resulted in depolymerization of tubulin, which then precipitated into paracrystalline inclusion bodies (**Figure 3A–C**), whereas untreated cells showed the characteristic fibrillar tubulin structure (**Figure S2**). Double labeling experiments showed that both FGF-2 (**Figure 3A**) and syndecan-1 (**Figure 3B**) strictly co-localized with the depolymerized tubulin; furthermore, that tubulin depolymerization completely hampered the nuclear translocation of both syndecan-1 and FGF-2. In contrast, FGFR-1 was not associated with tubulin, and its distribution was not affected by vinblastine treatment (**Figure 3C**). FGFR-1 and tubulin had distinct cytoplasmic localization and were completely independent of each other. These results show that the transport route of FGFR-1 differs from that of syndecan-1 and FGF-2, which both associate and co-precipitate with the depolymerized tubulin. Doxorubicin, known to arrest cells in the G_2_ phase of the cell cycle, almost completely inhibited the nuclear transport of syndecan-1. When cells were exposed to doxorubicin from the time of cell seeding, sporadic weak nuclear syndecan-1 staining could be observed in single scattered cells only (**Figure 3E**). In contrast, the untreated cells showed distinct nuclear staining of syndecan-1 (**Figure 3D**).

### The sub-cellular localization of exogenous FGF-2

**Figure 4:**
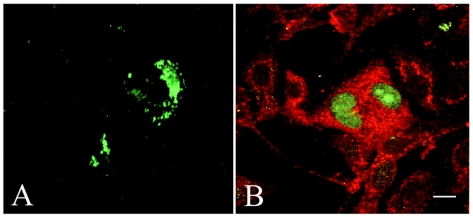
The sub-cellular localization of exogenously added FGF-2 differs from that of endogenous FGF2. In the STAV-AB cells, no nuclear accumulation of exogenously added Alexa 488-labeled FGF-2 could be observed 42 hours after seeding (A). In parallel, immunocytochemical staining (B) showed the presence of an endogenous pool of FGF-2 (*green*) in the nucleus, whereas syndecan-1 (*red*) remained cytoplasmic at this time point.

In order to see whether externally administered FGF-2 would translocate to the nucleus via a tubulin-syndecan-1 mediated transport route, Alexa-labeled FGF-2 was added exogenously to the cell cultures. The intracellular distribution of Alexa-labeled FGF-2 was restricted to the cytoplasm of mesothelioma STAV-AB cells up to 42 hours after seeding (**Figure 4A**). This time scale was chosen based on our previous observation on the time course of the nuclear translocation of syndecan-1 [15]. In parallel, double staining (**Figure 4B**) revealed a substantial pool of endogenous FGF-2 in the nucleus, whereas syndecan-1 remained cytoplasmic.

### Transport route and sub-cellular localization of newly synthesized syndecan-1

**Figure 5:**
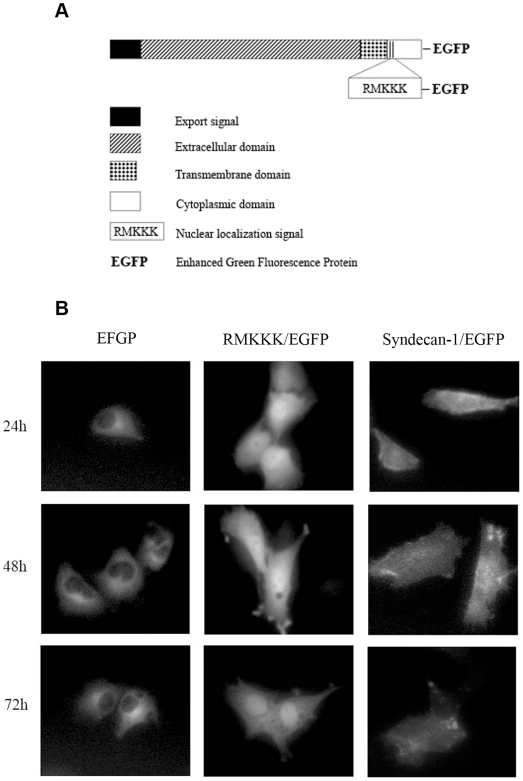
Schematic representation and sub-cellular localization of the syndecan-1/EGFP fusion proteins. The whole syndecan-1 molecule was fused to EGFP, whereas the truncated RMKKK/EGFP construct contained only the tubulin binding RMKKK motif, which acts as a nuclear localization signal. The figure indicates where in the cytoplasmic domain of syndecan-1 the RMKKK sequence is located (A). The sub-cellular localization of the syndecan-1/EGFP fusion proteins, after transfection into the B6FS cells were detected by fluorescence microscopy (B). EGFP-transfected control cells revealed only cytoplasmic reactivity at various time points (24–72 h) (B, *left column*). Distinct nuclear localization was seen in the RMKKK/EGFP transfected cells (B, *middle column*), whereas the syndecan-1/EGFP fusion protein revealed faint nuclear, cytoplasmic and focal cell membrane reactivities (B, *right column*).

**Figure 6:**
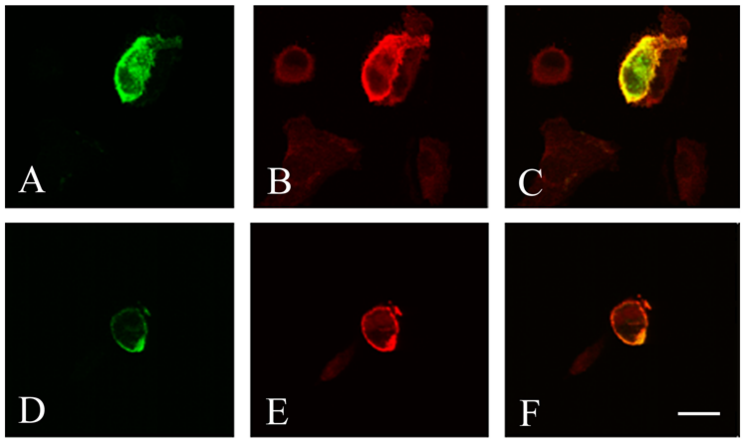
Immunocytochemical detection of the syndecan-1 ectodomain and HS chains in syndecan-1/EGFP transfectants. In the STAV-AB mesothelioma cells, cell membrane EGFP fluorescence appeared only in scattered cells 48 hours after transfection with syndecan-1/EGFP construct (A, D). The immunocytochemical detection of the syndecan-1 ectodomain (CD-138, Mouse monoclonal IgG1, detected by Goat anti-mouse IgG_1_ Alexa 568) is shown in (B), and the HS chains (mouse monoclonal IgM, clone 10E4, detected by Goat α-mouse IgM Alexa 568) in (E). The syndecan-1/EGFP fusion protein co-localized with the total amount of syndecan-1(C) and to some extent with the total amount of HS (F). *Bar*  = 20 µm.

The newly synthesized syndecan-1/EGFP and RMKKK/EGFP fusion proteins revealed signals of varying intensity and distinct sub-cellular distributions by fluorescence microscopy (**Figure 5**). The specificity of the signal was confirmed by immunocytochemical staining, using the CD138 antibody (**Figure 6**), which specifically recognizes the extracellular domain of syndecan-1, and verifies that functional syndecan-1 is being synthesized.

In the transfected B6FS cells, detectable amount of syndecan-1/EGFP fluorescence was seen already 6 hours after transfection, and the expression pattern changed over time. Cytoplasmic fluorescence was seen at all time points, but after 6–12 hours there was a clear perinuclear accumulation and after 24 hours syndecan-1 appeared in the nucleus (**Figure 5B**). Notably, the most prominent nuclear signal was obtained with the RMKKK/EGFP sequence, suggesting that this pentapeptide may act as a putative nuclear localization signal for syndecan-1 (**Figure 5B**). Such nuclear localization appeared in approximately 60% of the transfected cells, whereas no nuclear localization was observed in the corresponding EGFP controls (**Figure 5B**). Similar sub-cellular distribution was also seen in the other studied cell types, i.e. the STAV-AB and STAV-FCS malignant mesothelioma cell sub-lines (**Figure S3**). Accumulation of syndecan-1/EGFP in the cell membrane could only be observed in few scattered cells (**Figure 6A, D**). The EGFP fluorescence coincided with the syndecan-1 ectodomain (**Figure 6B, C**). Furthermore, cell surface reactivity to HS could be detected by the 10E4 antibody in the syndecan-1/EGFP transfected cells. HS co-localized with EGFP florescence at the cell membrane, revealing that the newly synthesized syndecan-1/EGFP fusion protein also carried HS chains (**Figure 6D–F**).

### Mutational analysis of the RMKKK sequence

**Figure 7:**
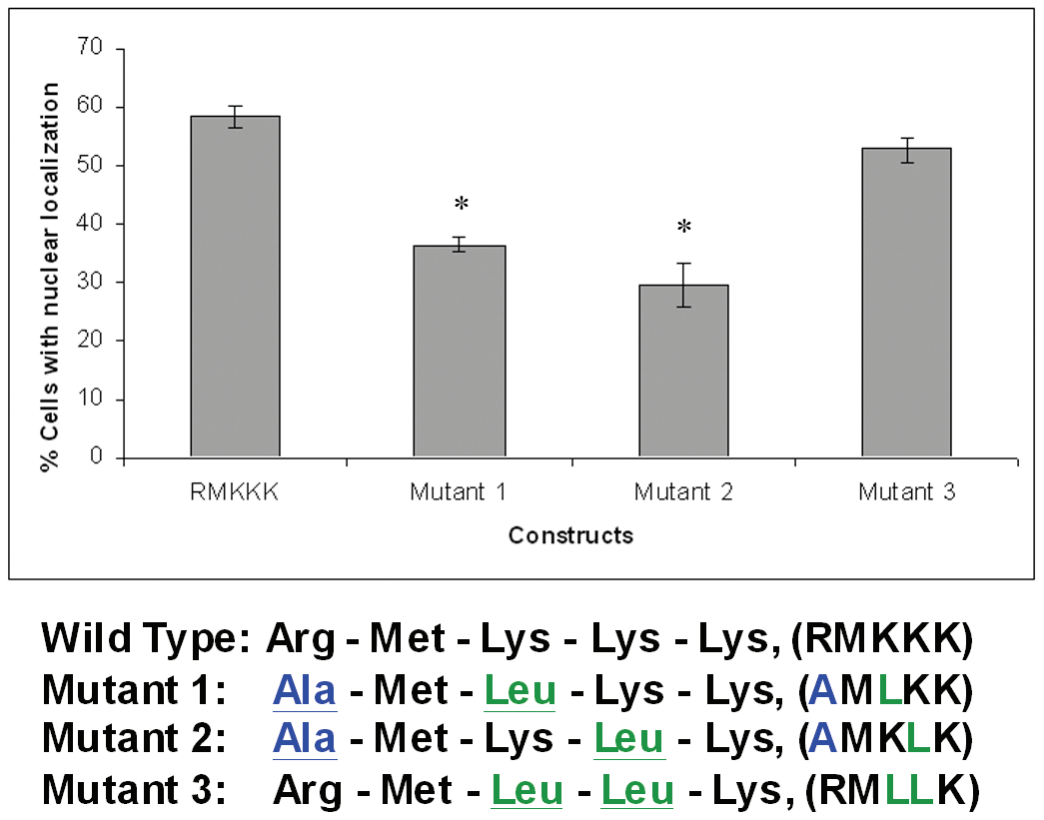
Site directed mutational analysis of the RMKKK sequence in B6FS cells. Mutants 1 and 2, but not mutant 3 showed a significant decrease in the proportion of cells with nuclear localization of the respective EGFP construct, when compared to the wild type RMKKK transfectants. Asterisks denote a statistically significant difference from RMKKK. Error bars show standard errors of the mean (SEM) of three independent experiments. Mutations are highlighted in color in the scheme of the mutants.

In cells transfected to express the RMKKK mutant 1 (**Ala** - Met - **Leu** - Lys – Lys, AMLKK) or mutant 2 (**Ala** - Met - Lys - **Leu** – Lys, AMKLK), both containing alanine instead of arginine, the proportion of cells with a nuclear positivity for EGFP signal was reduced by up to 50% compared to wide type RMKKK (**Figure 7**). These EGFP fusion proteins were evenly distributed over the entire cell, without accumulation in any sub-cellular compartment. The replacement of two of the three lysines with leucines, but retention of the arginine, in mutant 3 (Arg – Met -**Leu** - **Leu** – Lys, RMLLK), did not significantly affect the nuclear localization. These results indicate the essential role of the arginine residue for the nuclear translocation of the RMKKK motif.

## Discussion

Malignant mesothelioma and fibrosarcoma are aggressive tumors of mesenchymal origin, and they express a characteristic PG profile, in which syndecan-2 and -4 are the main cell-surface PGs [35], [36]. Syndecan-1 is less abundant at the surface of mesothelioma cells, and the most prominent syndecan-1 reactivity is seen in the nucleus and in the mitotic spindle during mitosis. Moreover, the accumulation of syndecan-1 in the nucleus is tubulin-dependent and precedes the cell membrane reactivity, which is seen later when the culture becomes confluent. This phenomenon has been observed in a series of both benign and malignant cells [15].

The co-localization of syndecan-1 with heparanase in cell nuclei suggests a simultaneous mechanism for the turnover of HS, indicating a regulatory importance of the nuclear syndecan-1. Similar nuclear accumulation occurs for growth factors, in particular FGF-2. In the mesothelioma cells we could detect a nuclear pool of syndecan-1 and FGF-2, whereas FGFR-1 remained exclusively perinuclear. In contrast to other cell types, this receptor never reached the nuclear compartment [25], [26], [27], [39]. Our results not only add to the growing literature showing a HS-mediated mechanism of nuclear translocation of FGF-2 [24], [40], but also identify a tubulin-mediated transport route for both syndecan-1 and FGF-2.

Immunoprecipitation experiments indicate that the co-localization of FGF-2 with syndecan-1 is associated with a physical interaction between the two proteins (**Figure 2**). We have recently proved by FACS analysis that syndecan-1 protein levels are increased 2-3 fold in these stable transfectants [36]. Our present data show that by overexpressing syndecan-1 the amount of nuclear FGF-2 increased approximately 2-fold, which provides evidence that syndecan-1 is needed for the nuclear transport of FGF-2. The presence of a greater amount of FGF-2 in the crude nuclear extract indicates that only part of the nuclear FGF-2 is bound to syndecan-1. This finding suggests that there also are other syndecan-1 independent routes for FGF-2 transport to the nucleus. Tubulin depolymerization prevents the nuclear transport of FGF-2 and syndecan-1 (**Figure 3A, B**), and the fact that both components co-localize with the depolymerized tubulin indicates stable associations between them. They thus seem to share a common tubulin-dependent transport mechanism. In contrast, no nuclear localization (**Figure 1D**) or co-localization with tubulin was observed for FGFR-1 (**Figure 3C**) pointing toward a divergent transport route for this molecule.

We have previously shown nuclear syndecan-1 in many other cell types besides malignant mesothelioma, including benign mesothelial cells, normal dermal fibroblasts, endothelial cells, adenocarcinomas and neuroblastoma cells [15]. In the present study, we show that the cytoplasmic RMKKK sequence of syndecan-1 is acting as a nuclear localization signal. This sequence corresponds to the characteristic short sequence of positively charged amino acids like lysine (K) and arginine (R) that defines a nuclear protein [41], [42]. It is located in the highly conserved C1 domain of syndecan-1, also known to be involved in the linkage to several cytoskeletal proteins, including tubulin, cortactin and ezrin [3]. Our findings show that this short motif of syndecan-1 is both necessary and sufficient for the nuclear translocation of a protein, as shown here fused to EGFP (**Figure 5**). Transfection with the RMKKK/EGFP construct results in a rapid accumulation of the fusion protein in the cell nucleus, whereas no such nuclear accumulation is observed in the control EGFP-transfected cells. Furthermore, replacement of the one arginine is sufficient to substantially decrease the nuclear accumulation of the mutated fusion proteins. The mutants 1 and 2 share a common arginine mutation and give a dramatic decrease of nuclear translocation, which indicates that arginine is most crucial for this function (**Figure 7**). However, the syndecan-1/EGFP construct, carrying the EGFP on the cytoplasmic tail, translocated to the nucleus much less efficiently than the native syndecan-1 or the RMKKK/EGFP construct. This indicates that the C-terminal region of syndecan-1 is important for the tubulin-mediated nuclear transport.

The EGFP fluorescence co-localizes both with newly synthesized syndecan-1 protein and HS at the cell membrane, revealing that the newly synthesized syndecan-1/EGFP fusion protein also carries HS chains (**Figure 6**), which is important for its GF binding capacity. To what extent the HS chains are themselves important for the nuclear translocation process is still an open and challenging question. Chen L *et al* have recently shown that heparanase overexpression or addition of recombinant heparansae decreases the nuclear syndecan-1 in a concentration-dependent manner, suggesting that the translocation is dependent to a significant extent upon the HS chains [43].

It has been suggested that the interaction of syndecan-1 with GFs on the cell surface may assist the internalization and intracellular trafficking of these factors. This mechanism is not completely elucidated, but several lines of evidence indicate the importance of HSPG in this process. Furthermore, GFs like FGF-2 and PDGF can act by triggering second messenger systems in addition to their effects on gene transcription [44]. Studies show that internalized FGF-2 survives longer periods of time in cells expressing HSPGs than in HSPG-deficient cells [45]. The majority of GF/GFR complexes on the cell surface get internalized and degraded in lysosomes, perhaps as part of the receptor-turnover pathway (for review see [46], [47]). The HSPG/GF complex might serve as a reservoir for GFs protecting them from degradation and allowing later entrance into the nucleus. However, such transport of exogenous FGF-2 from the cell surface to the nucleus could not be verified in the mesothelioma cells (**Figure 4A**). The finding that exogenously added GF never reached the nucleus in detectable amounts, contradicts the idea that this is a major route for FGF-2 present in the nucleus. In contrast, a substantial pool of endogenous FGF-2 is detected in the nucleus at the same time point by a parallel immunostaining (**Figure 4B**), suggesting an independent intracrine route, besides a tubulin/syndecan-1 mediated nuclear translocation.

Overexpression of syndecan-1 increased the amount of the nuclear FGF-2 in the crude nuclear extract and also co-immunoprecipitated together with the ectodomain of syndecan-1 (**Figure 2**), thereby emphasizing the role of syndecan-1 for this nuclear translocation.

Whether syndecan-1 follows an intracrine route or passes the cell membrane can not be answered from the present experimental results. The fact that syndecan-1 is seen in the nucleus before it accumulates in the cell membrane [15] to some extent supports the hypothesis of an intracrine loop. Since there is a strict co-localization of both N- and C-terminal portions of the syndecan-1 core protein with the HS side chains, the nuclear syndecan-1 seems to be the entire HSPG, which therefore must have been processed by the Golgi apparatus for HS modification before being transported to the nucleus.

It is plausible that this PG may be directed to different cellular compartments at different situations. Syndecan-1 gives faint immunocytochemical staining at the cell surface of the mesothelioma cells, detectable amounts being seen only at cell confluence. It may be that in the confluent cultures the shedding of syndecan-1 decreases, allowing it to accumulate on the cell surface [15]. The finding that FGF-2 and syndecan-1 often, but not always, co-localize in the nucleus, and that their expression levels vary during the experiments, also leaves the possibility of alternative routes for the nuclear translocation of these two components. This variability may also depend on a dynamic molecular switch consisting of subsequent association and dissociation events, where the activity of the HS moieties is modulated by the co-localizing heparanase. Once in the nucleus, however, syndecan-1 may well bind GFs, as shown by their tight co-localization. Furthermore, the activity of the heparanase may counteract the formation of GFs/syndecan-1 complexes, thereby regulating their possible functions.

The presence of syndecan-1 inside the nucleus raises many challenging questions remaining to be elucidated, including the nuclear targets of syndecan-1 and their possible downstream effects. Studies on such effects of nuclear syndecan-1 have been initiated in our laboratory.

## Supporting Information

Figure S1 Characteristic staining pattern of syndecan-1 in malignant cells. Immunocytochemical staining of syndecan-1 by CD138 antibody was performed in MCF-7 breast cancer (A, B), WART adenocarcinoma (C) and HTB-11 neuroblastoma (D) cells. Distinct nuclear syndecan-1 reactivity was seen in all cells. In addition to nuclear staining, a prominent perinuclear staining was seen in (B), distinct cell membrane staining at the cell-cell contact sites in (C), and cytoplasmic and cell membrane positivity in (D).
(1.48 MB TIF)

Figure S2 Tubulin structure in the mesothelioma cells. Sub-confluent the STAV-AB malignant mesothelioma cells were stained with primary antibody to α-tubulin (mouse monoclonal IgG1, Sigma T5168), and detected by green fluorescent secondary antibody (goat anti-mouse IgG (H+L), F(ab')2 Alexa 488, Molecular Probes A11017). Typical fibrillar tubulin structure was seen in the mesothelioma cells without vinblastine treatment.
(0.59 MB TIF)

Figure S3 Sub-cellular localization of syndecan-1/EGFP fusion proteins in the STAV-AB and STAV-FCS mesothelioma cell lines. The EGFP-transfected control cells displayed only cytoplasmic reactivity at various time points (24–72 h) (left column). Distinct nuclear localization was seen in the RMKKK/EGFP transfected cells (middle column), whereas the syndecan-1/EGFP fusion protein revealed faint nuclear, cytoplasmic and focal cell membrane reactivities (right column).
(0.83 MB TIF)
