# Supplementary figures and images for: Syndecan-1 and FGF-2, but Not FGF Receptor-1, Share a Common Transport Route and Co-Localize with Heparanase in the Nuclei of Mesenchymal Tumor Cells
Source: PLoS One. 2009 Oct 5;4(10):e7346. doi: 10.1371/journal.pone.0007346 (PMC2750749; doi:10.1371/journal.pone.0007346)

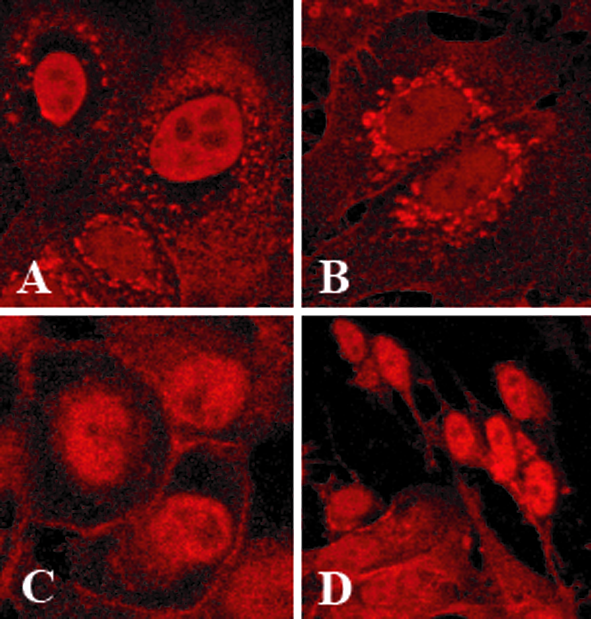

Supplement: Figure S1 — Characteristic staining pattern of syndecan-1 in malignant cells. Immunocytochemical staining of syndecan-1 by CD138 antibody was performed in MCF-7 breast cancer (A, B), WART adenocarcinoma (C) and HTB-11 neuroblastoma (D) cells. Distinct nuclear syndecan-1 reactivity was seen in all cells. In addition to nuclear staining, a prominent perinuclear staining was seen in (B), distinct cell membrane staining at the cell-cell contact sites in (C), and cytoplasmic and cell membrane positivity in (D). (1.48 MB TIF) [file pone.0007346.s001.tif]

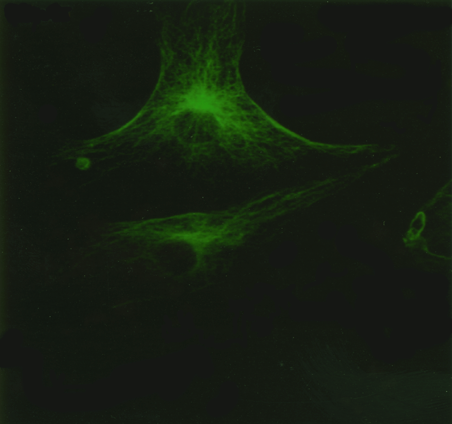

Supplement: Figure S2 — Tubulin structure in the mesothelioma cells. Sub-confluent the STAV-AB malignant mesothelioma cells were stained with primary antibody to α-tubulin (mouse monoclonal IgG1, Sigma T5168), and detected by green fluorescent secondary antibody (goat anti-mouse IgG (H+L), F(ab')2 Alexa 488, Molecular Probes A11017). Typical fibrillar tubulin structure was seen in the mesothelioma cells without vinblastine treatment. (0.59 MB TIF) [file pone.0007346.s002.tif]

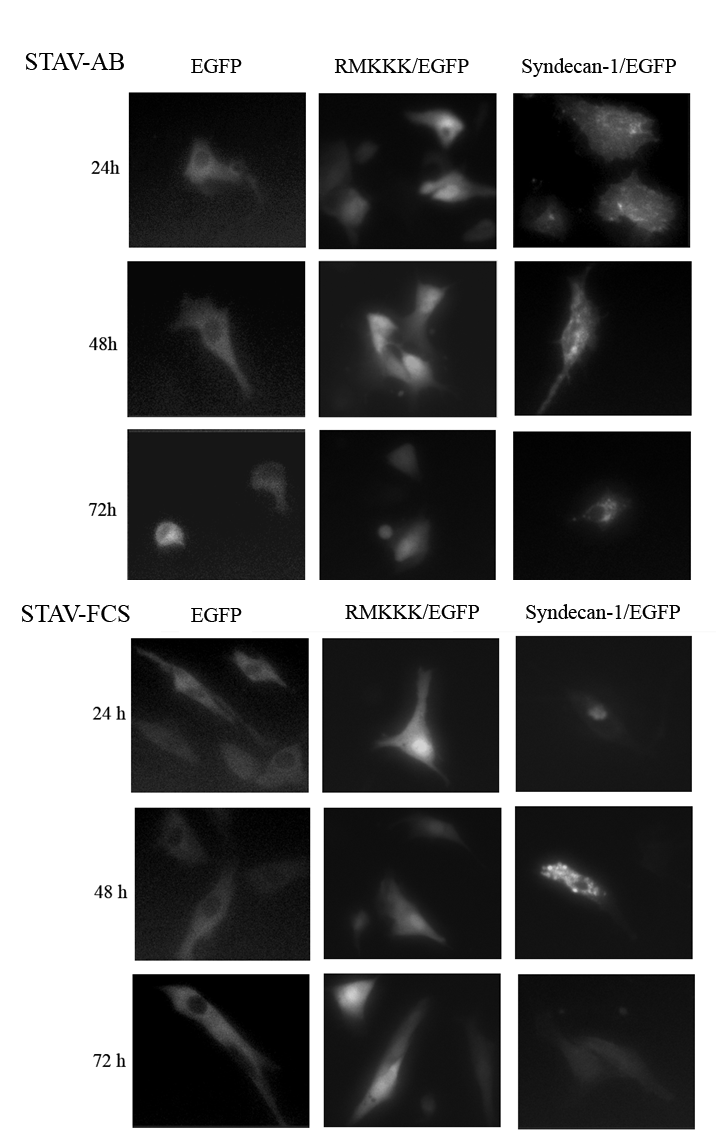

Supplement: Figure S3 — Sub-cellular localization of syndecan-1/EGFP fusion proteins in the STAV-AB and STAV-FCS mesothelioma cell lines. The EGFP-transfected control cells displayed only cytoplasmic reactivity at various time points (24–72 h) (left column). Distinct nuclear localization was seen in the RMKKK/EGFP transfected cells (middle column), whereas the syndecan-1/EGFP fusion protein revealed faint nuclear, cytoplasmic and focal cell membrane reactivities (right column). (0.83 MB TIF) [file pone.0007346.s003.tif]
